# Small incision reduction and external fixation for the treatment of delayed over fourteen days supracondylar humeral fractures in children

**DOI:** 10.3389/fped.2022.1039704

**Published:** 2022-11-02

**Authors:** Shuai Liu, YingYing Peng, JiaTong Liu, ZiXuan Ou, ZeZheng Wang, Saroj Rai, WeiFeng Lin, Xin Tang

**Affiliations:** ^1^ Pediatric Orthopedics Department, Wuxi 9th People's Hospital Affiliated to Soochow University, Wuxi, China; ^2^ Department of Orthopaedic, Wuhan Union Hospital, Tongji Medical College, Huazhong University of Science and Technology, Wuhan, China; ^3^ Department of Orthopaedics and Trauma Surgery, Karama Medical Center, Dubai, United Arab Emirates

**Keywords:** close reduction, minimally invasive technique, external fixation, delayed presentation, supracondylar humeral fracture, children

## Abstract

**Background:**

Supracondylar humeral fractures (SHF) are the most common type of fracture occurring at the distal humerus in children. In patients with delayed presentation of SHF, closed reduction is challenging to achieve with traditional reduction maneuvers. This study aimed to report the clinical results of pediatric SHF delayed over 14 days treated by closed reduction with a minimally invasive technique and external fixation and evaluate the efficacy of this technique.

**Methods:**

Between October 2010 and September 2018, children with delayed presentation of SHF over 14 days were retrospectively included in this study. The patients received closed reduction with a minimally invasive technique followed by external fixation. The demographics and radiographic data were collected. The Mayo Elbow Performance Score (MEPS) and the Flynn criteria were used to evaluate the clinical outcomes of treatments.

**Results:**

A total of 11 children (aged 4–13 years) with delayed presentation (range, 14–22 days) were recruited. They received surgery using closed reduction with a minimally invasive technique followed by external fixation. None of the surgery was done with the open method. After surgery, the patients' carrying angle returned to normal. The radiological union was evident in 8 to 12 weeks in all fractures without complications. Every patient had a good to excellent score on the MEPS and the Flynn criteria.

**Conclusions:**

The results of this series indicated a satisfactory outcome in children with delayed more than 14 days of supracondylar humeral fractures. The closed reduction with a minimally invasive technique followed by external fixation is an alternative treatment for such injury.

## Introduction

Supracondylar humeral fracture (SHF) is an extra-articular fracture that passes through the olecranon fossa, encompasses the distal humeral condyles, and is one of the most common elbow injuries in children (1). Delayed presentation of SHF is defined if the patient presents to the hospital after 2 days of injury (2, 3). Patients with delayed presentation (>1 week) may present with callus formation, union, nonunion and/or malunion with varying degrees of elbow deformity and dysfunction (4).

Treatment of delayed SHF aims to attain anatomic reduction, stable fixation, comprehensive function and good cosmetic results. However, there is no consensus regarding the appropriate treatment method for delayed SHF among orthopedic surgeons (5). Treatment includes closed reduction or open reduction with fixation. Closed reduction and percutaneous fixation has become the preferred treatment option for pediatric SHF but usually fails in patients presenting more than 7 days after injury (4). On the other hand, open reduction and internal fixation may cause iatrogenic neurovascular injury, wound infection, elbow stiffness and other complications (6).

This study aimed to report the clinical results of pediatric SHF delayed over 14 days treated by closed reduction with a minimally invasive technique and external fixation and evaluate the efficacy of this technique.

## Patients and methods

Children diagnosed with delayed SHF between October 2010 and September 2018 at the authors' hospital were retrospectively reviewed. The inclusion criteria were: (1) patients with delayed SHF more than 14 days after injury, (2) visible callus formation on the radiographs with failed manual reduction, (3) carrying angle of more than −15°, (4) availability of the complete clinical and radiological data, and (5) minimum follow up of 24 months. The exclusion criteria were: (1) patients with metabolic bone disease and (2) concomitant neurovascular injury needing exploratory surgery. Demographic data included age, gender, Gartland classification and time between injury and surgical intervention. All the patients' parents or legal guardians were fully informed of the surgical procedure and gave consent to be included in the study. It was informed that closed reduction might not be achieved and minimally invasive incision might be necessary. All patients were operated on by the same surgical team as per the standard protocol. This study was approved by the ethical review board of the corresponding author's institution.

### Surgical technique

**Figure 1:**
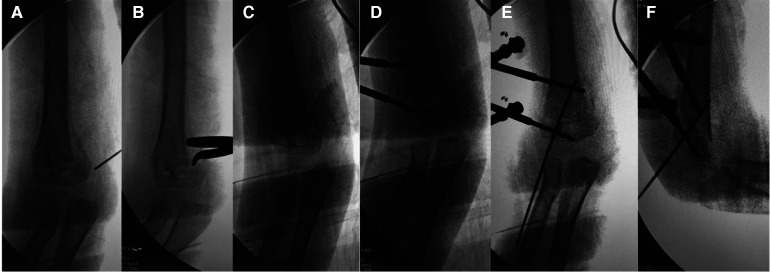
C-arm image during the surgical procedure of a 5-year-old boy with delayed supracondylar fracture of right humerus: (**A**) the fracture line with callus was located with a needle; (**B**) lateral, anterior, and posterior calluses were removed from the humerus horizontally along the fracture line with the hemostat; (**C**) manual reduction of fractures was performed to correct the abnormal carrying angle; (**D**) external fixation with two schanz pins was used; (**E**) placement of K-wire was served as a de-rotational wire to stabilize the fracture; (**F**) lateral view of the elbow after fixation.

All the procedure was performed under general anesthesia. The patient was positioned supine with the injured extremity close to the edge of the operating table. The fracture line with the callus was located under fluoroscopy (Figure 1A). A 5 mm skin incision was made from the medial aspect of the humerus, closed to the fracture line with callus and guided by the intraoperative fluoroscopy. A hemostat was inserted, and soft tissue was mobilized away from the callus. Lateral, anterior, and posterior calluses were removed from the humerus horizontally along the fracture line with the hemostat in order to loosen the fracture (Figure 1B). Manual reduction of fractures was performed in order to correct the abnormal carrying angle, rotation and shortening after the fracture was loosened (Figure 1C).

External fixation was used for patients as per the technique reported by Slongo T et al. (7) The first Schanz pin (3.0–4.0 mm) was placed perpendicular to the longitudinal axis of the proximal humerus and buried in the medial cortex, keeping 2 cm above the fracture line. The second pin was placed perpendicular to the longitudinal axis of the distal fragment and parallel to the elbow joint, keeping 1–2 cm below the fracture line (Figure 1D). It was regarded as an adequate and stable reduction without malrotation following manual reduction when these 2 pins became parallel. A 1.5–2.0 mm K-wire was passed retrograde from the lateral epicondyle crossing the fracture line as a de-rotational wire (Figure 1E,F). The stability of the fixation was then tested in maximum flexion and extension, and a check x-ray was obtained. The surgical procedure of a typical case is presented in Figure 1.

### Postoperative care and follow-up

**Figure 2:**
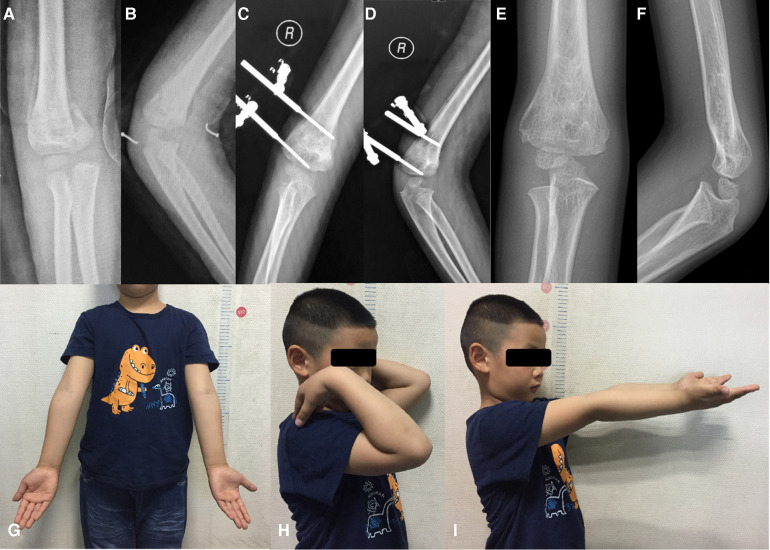
(**A**) anteroposterior and (**B**) lateral radiographs of the 5-year-old boy with delayed supracondylar fracture of right humerus; (**C**) anteroposterior and (**D**) lateral radiographs at 6 weeks post-operation; (**E**) anteroposterior and (**F**) lateral radiographs at 12 months post-operation; and the follow-up in 24 months after surgery show excellent cosmetic results (**G**) and the functional appearance (**H,I**).

All patients were discharged 2–3 days after surgery without a cast. Free range of motion with non-weight-bearing was allowed 48 h post-operation. The external fixation was removed 6 weeks postoperatively. Every patient returned for clinical and radiographic evaluations at 6 weeks, 12 weeks, 6 months, 12 months and 24 months. The elbow joint function was evaluated with the Mayo Elbow Performance Score (MEPS) and the criteria of Flynn at the last follow-up (8, 9). The typical case in fellow up was presented in Figure 2.

## Results

A total of 11 patients (8 boys and 3 girls) with an average age of 7 years (range, 4–13 years) were included in this study. Closed reduction with a minimally invasive technique achieved a satisfactory reduction in all patients. All cases were classified as Gartland type III and underwent surgery with an average of 17.7 days after injury (Table 1). The average duration of the surgery was 56 min (range, 50–65 min). The radiological union was evident in 8 to 12 weeks in all fractures. No complications such as Volkmann ischemic contracture, infection, nonunion, myositis ossificans, iatrogenic neurological injuries or residual vascular deficits were noted. The carrying angle difference between fractured and uninjured sides was less than 4°, and cosmetic results in all patients were excellent (Table 2). At the last follow-up, all patients reported 90 points or more on MEPS and good to excellent outcomes on Flynn criteria (Table 3). The therapeutic effect was satisfactory in all patients. Neither revision surgery after the initial fixation nor change in muscle power in the injured limb relative to the uninjured limb was reported at the last follow-up.

**Table 1:** Demographics of patients.

Serial No.	Age (years)	Gender	Gartland classification	Time between injury and surgical intervention (days)
1	7	F	type III	20
2	6	M	type III	16
3	6	F	type III	18
4	4	M	type III	17
5	13	M	type III	15
6	5	M	type III	21
7	5	M	type III	22
8	7	F	type III	17
9	8	M	type III	16
10	9	M	type III	19
11	12	M	type III	14

**Table 2:** Carrying angle of injured and uninjured sides before and after the operation.

Serial No.	Carrying angle of the operated side before the operation	Carrying angle of the operated side after the operation	Carrying angle of the uninjured side	Carrying angle difference between fractured and uninjured sides after the operation
1	−23°	13°	12°	1°
2	−18°	13°	11°	2°
3	−15°	15°	13°	2°
4	−15°	14°	12°	2°
5	−38°	8°	10°	−2°
6	−22°	10°	12°	−2°
7	−20°	8°	5°	3°
8	−15°	12°	13°	−1°
9	−17°	14°	11°	3°
10	−16°	15°	15°	0°
11	−16°	14°	10°	4°

**Table 3:** Perioperative and follow-up data.

Serial No.	Operation duration (min)	Time to union (weeks)	Follow-up time (months)	Flynn	MEPS
1	56	12	40	Excellent	95
2	60	10	34	Excellent	95
3	52	10	40	Excellent	95
4	50	8	52	good	90
5	65	12	70	Excellent	90
6	55	9	41	Excellent	95
7	54	8	36	Excellent	95
8	57	10	27	good	95
9	59	10	27	good	95
10	52	10	26	Excellent	100
11	56	12	27	good	95

MEPS, mayo elbow performance score.

## Discussion

To our best knowledge, this was the first study reporting the outcome of pediatric supracondylar humerus fractures treated with closed or mini-open reduction and external fixation after 14 days of injury. SHF accounts for 55% to 80% of total elbow fractures in children and up to two-thirds of pediatric elbow injuries require hospitalization (10). This type of fracture usually occurs as a result of a fall from height and the incidence is estimated to be 177.3 per 100,000 (11). Delayed presentation of SHF is defined if the patient presents to the hospital after 2 days of injury in developed countries (4). Prabhakar P et al. reported that surgical treatment of low-severity Gartland type III SHFs might be delayed without increasing surgical time and reduction difficulty, but only if the delay time was about 18.5 h, which was hardly a delay in developing countries (12). Silva M et al. showed that anatomic reduction of type II humeral supracondylar fractures could be achieved probably even when closed reduction and percutaneous pinning was performed 7 days after the original injury, but the risk of avascular necrosis of the humeral trochlea must be considered (13). In developing countries, the delayed duration always exceeds 7 days, and the reasons for a delay in interventions are quackery, lack of medical facilities, cost, poor economic status, lack of awareness, delayed referral from the rural hospital, fear of surgery, and various indigenous forms of treatment, which bring difficulty for closed reduction (14–16).

Optimum treatment of SHF is essential in order to avoid serious complications. It is well recognized that the Gartland type III and type IV fractures should be treated surgically (17). To date, closed reduction and percutaneous pinning is the gold standard for all displaced fractures (10). The advantages of closed reduction are the preservation of blood supply to the fracture site, shortening of hospital stays and reduction of risk of infection (18). However, controversy remains with regard to the timing of emergency reduction, whether reduction can be safely delayed, the adequate reduction technique, the risk/benefit ratio of open reduction and the long-term consequences of a cubitus varus deformity (19). It is a challenge for surgeons to improve the success rate of closed reduction, especially for delayed supracondylar humeral fractures. Displaced supracondylar fractures had been traditionally treated as surgical emergencies for the reason that delayed surgery often required open reduction rather than closed reduction (20). In the case of delayed presentation, especially for Gartland type III fractures which is a statistically significant independent risk factor for closed reduction failure, the probability of fracture swelling is significantly increased, for which open reduction is needed to achieve better outcomes and avoid complications such as iatrogenic neurovascular injuries, stiffness, delayed union, malunion and nonunion (14, 15, 21, 22). A meta-analysis conducted by Farrow L et al. showed that there was no statistically significant difference in the risk of complications between immediate and 91-h delayed treatment for patients with SHFs undergoing open reduction (23). However, all the patients in this study were Gartland type III fractures and were delayed over 14 days. All achieved a satisfactory result with reduction with a minimally invasive technique.

The formation of intraperiosteal bone begins immediately after the fracture, but proliferative activity in the cells appears to cease before 2 weeks. By the time the endochondral process has reached the stage of chondrogenesis, a large number of woven bone forms near the fracture site. Once the fracture coalesces through the bone-bridging gap, the callus (composed entirely of woven bone) remodels to form a mechanically capable layered structure (24). Callus formation occurs even faster in children, which is why closed reduction over 14 days often fails and open reduction is the only option left. Close reduction avoids complications related to open reduction, such as wound infection or elbow stiffness (18, 25, 26). However, the closed reduction could not be achieved in a single patient presenting more than 7 days after injury in Tiwari A et al.'s study because closed reduction and cast fixation were not feasible in late-presenting SHFs. The injury is usually accompanied by severe swelling that prevents rapid and safe flexion, and soft tissue scabbed at the end of the first week precludes reduction of the fracture (4). All the patients achieved a satisfactory reduction using the minimally invasive technique in this study. Traditional K-wires fixation of SHF was not performed in this series because without removing the callus from the fracture site, the fixation could not be stable only by K-wires. Instead, the external fixator could provide better stability than K-wires. Sufficient stability following the use of an external fixator allowing an early functional exercise was indicated by good to excellent functional outcomes. Also, none of the cases underwent revision surgery in our series.

The carrying angle of the elbow is used to assess varus or valgus deformity (19). The patients' carrying angles in this study were over −15° pre-operation, with a high risk of cubitus varus deformity that might lead to a second corrective osteotomy. After the operation, the carrying angle difference between fractured and uninjured sides was less than 4°, indicating that the reduction of fracture by minimally invasive technique and application of external fixation corrected the preoperative cubitus varus effectively. Masumbuko CK et al. showed that delaying surgery for more than seven days resulted in reduced elbow range of motion (27). In contrast, midterm follow-up results of the elbow function were satisfactory in our study. The advantages of external fixation include stable fixation avoiding delayed healing and early mobilization, which may contribute to good functional results (7, 28). This method reduced the risk of complications of open reduction, such as neurovascular injury, elbow stiffness, wound infection and ugly scarring, as well as complications due to unsatisfactory closed reduction, including triceps fibrilization and limited elbow mobility.

Limitations of this study were the small number of cases, failure to follow-up until the closure of physes, and its retrospective nature. This case series could not provide a control group because none of the parents or guardians chose to wait for a long-term outcome with a high risk of cubitus varus. They all chose a one-stage procedure in view of carrying angle >−15°.

## Conclusions

The results of this series indicated a satisfactory outcome in children with delayed more than 14 days of supracondylar humeral fractures. The closed reduction with a minimally invasive technique followed by external fixation is an alternative treatment for such kind of injury.

## Data Availability

The original contributions presented in the study are included in the article/Supplementary Material, further inquiries can be directed to the corresponding author/s.
